# Recurrence‐associated gene signature optimizes recurrence‐free survival prediction of colorectal cancer

**DOI:** 10.1002/1878-0261.12117

**Published:** 2017-09-23

**Authors:** Xianglong Tian, Xiaoqiang Zhu, Tingting Yan, Chenyang Yu, Chaoqin Shen, Ye Hu, Jie Hong, Haoyan Chen, Jing‐Yuan Fang

**Affiliations:** ^1^ Division of Gastroenterology and Hepatology Key Laboratory of Gastroenterology and Hepatology Ministry of Health State Key Laboratory for Oncogenes and Related Genes Renji Hospital School of Medicine Shanghai Institute of Digestive Disease Shanghai JiaoTong University China

**Keywords:** colorectal cancer, gene signature, prognosis, recurrence‐free survival

## Abstract

High throughput gene expression profiling has showed great promise in providing insight into molecular mechanisms. Metastasis‐related mRNAs may potentially enrich genes with the ability to predict cancer recurrence, therefore we attempted to build a recurrence‐associated gene signature to improve prognostic prediction of colorectal cancer (CRC). We identified 2848 differentially expressed mRNAs by analyzing CRC tissues with or without metastasis. For the selection of prognostic genes, a LASSO Cox regression model (least absolute shrinkage and selection operator method) was employed. Using this method, a 13‐mRNA signature was identified and then validated in two independent Gene Expression Omnibus cohorts. This classifier could successfully discriminate the high‐risk patients in discovery cohort [hazard ratio (HR) = 5.27, 95% confidence interval (CI) 2.30–12.08, *P* < 0.0001). Analysis in two independent cohorts yielded consistent results (GSE14333: HR = 4.55, 95% CI 2.18–9.508, *P* < 0.0001; GSE33113: HR = 3.26, 95% CI 2.16–9.16, *P* = 0.0176). Further analysis revealed that the prognostic value of this signature was independent of tumor stage, postoperative chemotherapy and somatic mutation. Receiver operating characteristic (ROC) analysis showed that the area under ROC curve of this signature was 0.8861 and 0.8157 in the discovery and validation cohort, respectively. A nomogram was constructed for clinicians, and did well in the calibration plots. Furthermore, this 13‐mRNA signature outperformed other known gene signatures, including oncotypeDX colon cancer assay. Single‐sample gene‐set enrichment analysis revealed that a group of pathways related to drug resistance, cancer metastasis and stemness were significantly enriched in the high‐risk patients. In conclusion, this 13‐mRNA signature may be a useful tool for prognostic evaluation and will facilitate personalized management of CRC patients.

**Abbreviations**
AJCC: American Joint Committee on Cancer
AUC: area under receiver operating characteristic
CI: confidence interval
CRC: colorectal cancer
DFS: disease‐free survival
GEO: Gene Expression Ominus
HR: hazard ratio
LASSO: least absolute shrinkage and selection operator method
RFS: recurrence‐free survival
ROC: receiver operating characteristic
ssGSEA: single‐sample gene‐set enrichment analysis
TNM: TNM Classification of Malignant Tumours

## 1 Introduction

Colorectal cancer (CRC) is one of the most common cancers worldwide. It ranks as the fourth leading cause of cancer death after lung, liver, and stomach cancer (Ferlay *et al*., 2015). Currently, surgery and chemotherapy are the most common treatments, and the treatment selection is mainly based on the tumor stage. The American Joint Committee (AJCC) staging system on cancer has been widely used for cancer management clinically (Kawaguchi *et al*., 2013; Marrelli *et al*., 2012). However, the TNM Classification of Malignant Tumours staging method (TNM; AJCC 6th edn) cannot provide accurate information to clinicians for predicting patient survival time. The higher TNM stage is generally associated with a poorer outcome. However, the prognosis for stage IIb patients is significantly worse than for those with stage IIIa (O'Connell *et al*., 2004). Tumor stage can help guide chemotherapy for CRC patients, and adjuvant chemotherapy is accepted as standard regimen for these stage III patients (NIH Consensus Conference, 1990), but it is still debatable for those with stage II (Benson *et al*., 2004; Figueredo *et al*., 2008; Ratkin, 1997), indicating that the TNM staging system is not totally recommended for the management of CRC. These limitations have prompted a search for new biomarkers for discrimination of high‐risk patients to improve personalized cancer care.

CRC is of a high heterogeneity, originating from complex interactions between environmental and genetic factors (Lichtenstein *et al*., 2000). Some critical genes and/or associated signaling pathways, such as chromosomal instability, RAS, Wnt, and other pathways (Malumbres and Barbacid, 2003; Pino and Chung, 2010; Sparks *et al*., 1998) are implicated in the initiation, progression, and metastasis of CRC. Great efforts have been made to identify the molecular markers for prognosis prediction. However, a systemic analysis has found conflicting evidence as to the prognostic significance of genes commonly implicated in the pathogenesis of CRC (Anwar *et al*., 2004).

In recent years, many studies have focused on gene expression profiles in CRC; these have shown great promise for predicting prognosis in individual patients. A test approved by the US Food and Drug Administration (MammaPrint; Agendia, Amsterdam, the Netherlands) has been successfully developed for prognostic prediction in breast cancer (Glas *et al*., 2006; van ‘t Veer *et al*., 2002). Several gene signatures have also been established to distinguish the prognosis of patients beyond the CRC clinicopathological features; however, most of them are not used clinically (Agesen *et al*., 2012; Jensen *et al*., 2015; O'Connell *et al*., 2010; Oh *et al*., 2012; Schell *et al*., 2016; van der Stok *et al*., 2016). Thus, identifying a more powerful and practical gene signature for prognosis prediction is of great clinical significance.

We mined previously published gene expression microarray data from the Gene Expression Omnibus (GEO), and conducted mRNA profiling on large cohorts of CRC patients. The differentially expressed mRNAs were identified by analyzing the metastasized and non‐metastasized CRC tissues. According to the TNM staging system (AJCC 6th edn), non‐metastasized and metastasized patients belonged to different tumor stages and demonstrated significantly different outcomes (O'Connell *et al*., 2004). Thus these metastasis‐related expression changes might be enriched with genes with potential prognostic predictive value, useful for developing a gene signature for predication of recurrence of these mRNAs. For selection of prognostic genes, the least absolute shrinkage and selection operator method (LASSO) has been extensively applied in high‐dimensional microarray data (Gui and Li, 2005; Tibshirani, 1997; Zhang *et al*., 2013). By this way, we identified a 13‐mRNA signature in discovery set GSE17536 to predict recurrence‐free survival (RFS) for patients with CRC. RFS was defined as the incidence of recurrence after resection, which was also called disease‐free survival (DFS) (Jorissen *et al*., 2009; Kemper *et al*., 2012; Smith *et al*., 2010). We validated it in another two independent cohorts (GSE14333 and GSE33113) and assessed the prognostic value of this gene signature in discovery and validation datasets. Furthermore, a comparison was made between our 13‐mRNA signature and other three important gene signatures, including OncotypeDX colon cancer assay (Srivastava *et al*., 2014).

## 2 Materials and methods

### 2.1 CRC gene expression data

CRC gene expression data and corresponding clinical data used in this study are available on arrayexpress (http://www.ebi.ac.uk/arrayexpress/) and GEO (https://www.ncbi.nlm.nih.gov/geo/). All data with raw data CEL files were under the same chip platform (Affymetrix human genome u133 plus 2.0 chips). The raw data were downloaded and normalized using a robust multi‐array averaging method (Irizarry *et al*., 2003). We processed the Affymetrix data using ‘affy’ and ‘affycoretools’ packages of r software (version 3.3.1, R Foundation for Statistical Computing Vienna, Austria). This well‐defined process consisted of the following steps: first, importing the ‘raw’ data in.CEL format and the associated clinical information; secondly, summarizing the expression values for each probe set; the last step included background correction, normalization and summarizing. After excluding the samples without valuable clinical survival information, 556 patients in four datasets were used in this study, including GSE64256 (*n* = 125), GSE17536 (*n* = 145), GSE14333 (*n* = 197), GSE33113 (*n* = 89) (see Tables [Link], [Link], [Link]). Tumors in GSE14333 were recorded with Dukes’ stages, which were converted to AJCC stages based on the AJCC Colon and Rectum Cancer staging, 7th Edition, in order to maintain consistency with other datasets. GSE64256 dataset was used to identify the differentially expressed mRNAs between 26 metastasized samples and 99 non‐metastasized samples. Dataset GSE17536 was used as the discovery set to screen out the prognostic gene signature from the differentially expressed mRNAs. The gene signature was then validated in GSE14333 and GSE33113 datasets. The flowchart of this study was depicted in Fig. S1.

### 2.2 Identification and validation of the prognostic gene signature

At first, to construct the prognostic gene signature from the metastasis‐related mRNAs, the ‘limma’ package of r software (version 3.3.1) was used to generate the differentially expressed mRNAs whose parameter *P*‐value was less than 0.05 between metastasized samples and non‐metastasized samples in GSE64256. Then, r software (version 3.3.1) and the ‘glmnet’ package (R Foundation for Statistical Computing, Vienna, Austria) were used to perform the LASSO Cox regression model analysis in the discovery dataset GSE17536. The penalized Cox regression model with LASSO penalty was used to achieve shrinkage and variable selection simultaneously, and the optimal values of the penalty parameter lambda were determined through 10‐times cross‐validations (Goeman, 2010; Tibshirani, 1997). Based on the optimal lambda value, a list of prognostic genes with associated coefficients was screened out from the metastasis‐related mRNAs based on the gene expression profiling and RFS data. The risk score for each patient was then calculated based on the expression level of each prognostic mRNA and its associated coefficient. The patients in each dataset were split into a low‐risk and a high‐risk group according to the median risk score. The median value of the risk score was set as the cut‐off, since its clinical application is easy. Finally, the Kaplan–Meier estimator and the log‐rank test were introduced to assess RFS differences between the low‐risk and high‐risk groups. The gene signature was validated in two independent datasets. The risk scores were calculated using the same formula as in the discovery set. Each dataset was divided into two risk groups based on the median risk score and the RFS differences were analyzed as above.

### 2.3 Statistical analysis

Univariable and multivariable Cox regression were performed to investigate whether this gene signature was independent of age, gender and tumor stage. Receiver operating characteristic (ROC) analysis was used to assess the sensitivity and specificity of the survival prediction based on the multi‐mRNA risk score, tumor stage, combined model of risk score and tumor stage, and prognostic indexes of other gene signatures. An area under ROC curve (AUC) was used as a measure of the accuracy in diagnostic tests (Bunger and Mallet, 2016). We adopted the ‘pROC’ package for ROC analysis, and the method ‘delong’ was used to test the significance of differences between the ROC curves. For ROC analysis, it was necessary to exclude patients who had not had a recurrence at the time of the last follow‐up and in whom RFS duration was less than the median RFS. The remaining patients were classified into two subgroups based on the median RFS (Kang *et al*., 2012).

Survival times of patients were from the date of surgery to the time of recurrence or the date on which data were taken, based on the method of Kaplan–Meier. The curves were analyzed using the log rank test. A *P*‐value less than 0.05 was set as the significant difference for all the Cox regression analyses, log‐rank tests and ROC analyses.

### 2.4 Construction of nomogram

The nomogram and calibration plots were generated using the ‘rms’ package of r software (version 3.3.1). The predictive accuracy of a nomogram was assessed by a concordance index which investigated the level of consistency between the actual observed outcome frequencies and predicted probabilities (Wang *et al*., 2013). After the construction of nomogram model, cross‐validation was performed to address model overfitting; a bootstrap resampling method was adopted to generate the confidence interval (CI) for concordance indexes (Pencina and D'Agostino, 2004; Wang *et al*., 2013). A calibration plot was used to visualize the performance of the nomogram. Nomogram‐predicted recurrence and observed outcome were plotted on the *x*‐axis and *y*‐axis, respectively; the 45° line represented the best prediction.

### 2.5 Gene set enrichment analysis

To identify the differentially expressed gene sets between the low‐risk and high‐risk subgroups, single sample gene set enrichment analysis (ssGSEA) was performed. Enrichment scores in each sample were calculated using the ‘GSVA’ package of r software (version 3.3.1) and its ssGSEA method (http://www.bioconductor.org) (Hanzelmann *et al*., 2013). The enrichment score represented the degree of absolute enrichment of a certain gene set in each sample within a dataset (Barbie *et al*., 2009; Subramanian *et al*., 2005). The risk‐associated gene sets (adjusted *P*‐value < 0.001) were identified for further analysis. For correlation analysis, ‘corrplot’ package was used, and the correlation coefficients, CI and *P*‐values were calculated using r software.

## 3 Results

### 3.1 Development and validation of prognostic 13‐mRNA signature

A set of 2848 differentially expressed mRNAs between metastasized and non‐metastasized tumors was identified from dataset GSE64256. LASSO Cox regression model was applied for further analysis of these 2848 genes in the discovery set GSE17536 (see Fig. S2). We identified a 13‐mRNA signature that was significantly correlated with RFS in CRC patients. Table S4 shows a list of probes with associated coefficients which were generated from the LASSO analysis.

The risk score for each patient was calculated based on the expression levels of all 13 genes in the multivariate model and their associated coefficients (see Table S5). Among the 13‐mRNAs, 11 genes had positive coefficients – THBS2, CAV2, SCG2, SLC6A1, SAV1, EZ6L2, ERO1A, RAB3B, OBSL1, CD109, and PTPN14. The coefficients for the other two genes (MRPL35, LRPAP1) were negative. For the CRC patients, the higher risk score meant a poorer prognosis; thus the higher expression levels of genes with a positive weighting coefficient indicated higher risk scores, and an increased risk of recurrence. Conversely, the higher expression levels of genes with a negative coefficient were associated with a better outcome.

**Figure 1:**
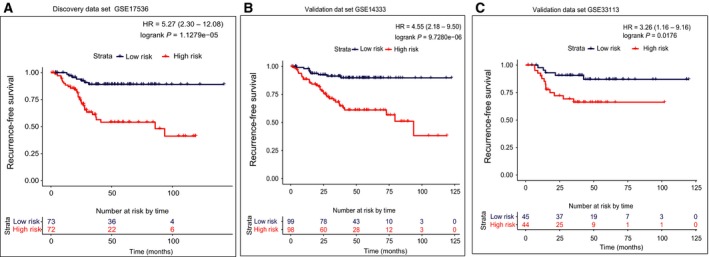
Kaplan–Meier estimates of the patients’ recurrence using the 13‐mRNA signature. The Kaplan–Meier plots were used to visualize the patients’ recurrence probabilities for the low‐risk versus high‐risk group of patients based on the median risk score from corresponding GEO datasets. (A) Kaplan–Meier curves for discovery dataset GSE17536 patients (*n *= 145). (B) Kaplan–Meier curves for GSE14333 patients (*n *= 197). (C) Kaplan–Meier curves for GSE33113 patients (*n *= 89). The tick marks on the Kaplan–Meier curves represent the subjects studied. The differences between the two curves were determined by the two‐sided log‐rank test.

**Figure 2:**
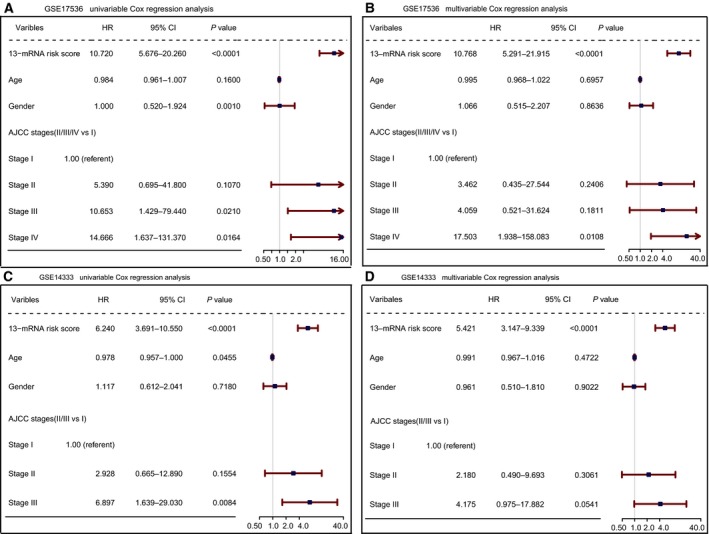
Forest plot summary of analyses of recurrence‐free survival (RFS). Univariable and multivariable analyses of the 13‐mRNA risk score, age, gender, and tumor stage on GSE17536 (A,B) and GSE14333 (C,D) datasets. The blue squares on the transverse lines represent the hazard ratio (HR), and the red transverse lines represent 95% CI. Risk score and age are continuous variables, gender and tumor stage are discontinuous variables.

The 13‐mRNA signature risk score for each patient was calculated in the discovery set GSE17536 (min: 13.18, median: 13.98, max: 15.48). In survival analysis, a dichotomous score was adopted. The patients were divided into a low‐risk group (*n* = 73) and a high‐risk group (*n* = 72) based on the median risk score. Patients in the high‐risk group demonstrated a worse outcome compared with those in the low‐risk group (HR = 5.27, 95% CI 2.30–12.08, *P *<* *0.0001) (Fig. 1A). The univariable and multivariable Cox regression analyses also showed that the 13‐mRNA risk score was significantly associated with RFS as a continuous variable (*P *<* *0.0001) (Fig. 2A,B). The distribution of risk score, the recurrence status of the CRC patients, and the mRNA expression profiling were analyzed and the results showed that significantly more patients had a recurrence in the high‐risk group than in the low‐risk group, and the expression levels of genes with positive coefficients were higher in high‐risk patients (Fig. S3).

The efficacy of the 13‐mRNA signature for prognosis prediction of CRC patients was further validated in two independent datasets (GSE14333 and GSE33113). Patients were classified into a high‐risk and a low‐risk group using the same risk score‐based classifier; the median risk score in each dataset was taken as the cut‐off value. Consistent with the findings described above, high‐risk patients in the GSE14333 cohort had a significantly shorter median RFS compared with low‐risk patients (HR = 4.55, 95% CI 2.18–9.50, *P* < 0.0001) (Fig. 1B). Analysis in the GSE33113 dataset produced similar results (HR = 3.26, 95% CI 1.16–9.16, *P* = 0.0176) (Fig. 1C). The univariable and multivariable Cox regression analyses also showed that the association of 13‐mRNA risk score with RFS was statistically significant as a continuous variable in the GSE14333 cohort (Fig. 2C,D).

We also performed a Chi‐square test to investigate whether the recurrence status was associated with risk stratification (low‐risk group versus high‐risk group). Results showed that the *P*‐values in all three cohorts were less than 0.05. Moreover, more samples in the patients with recurrence fell into the high‐risk group, in which the range and median of RFS were shorter than that in low‐risk group (Table S2).

### 3.2 Prognostic value of the 13‐mRNA signature

**Figure 3:**
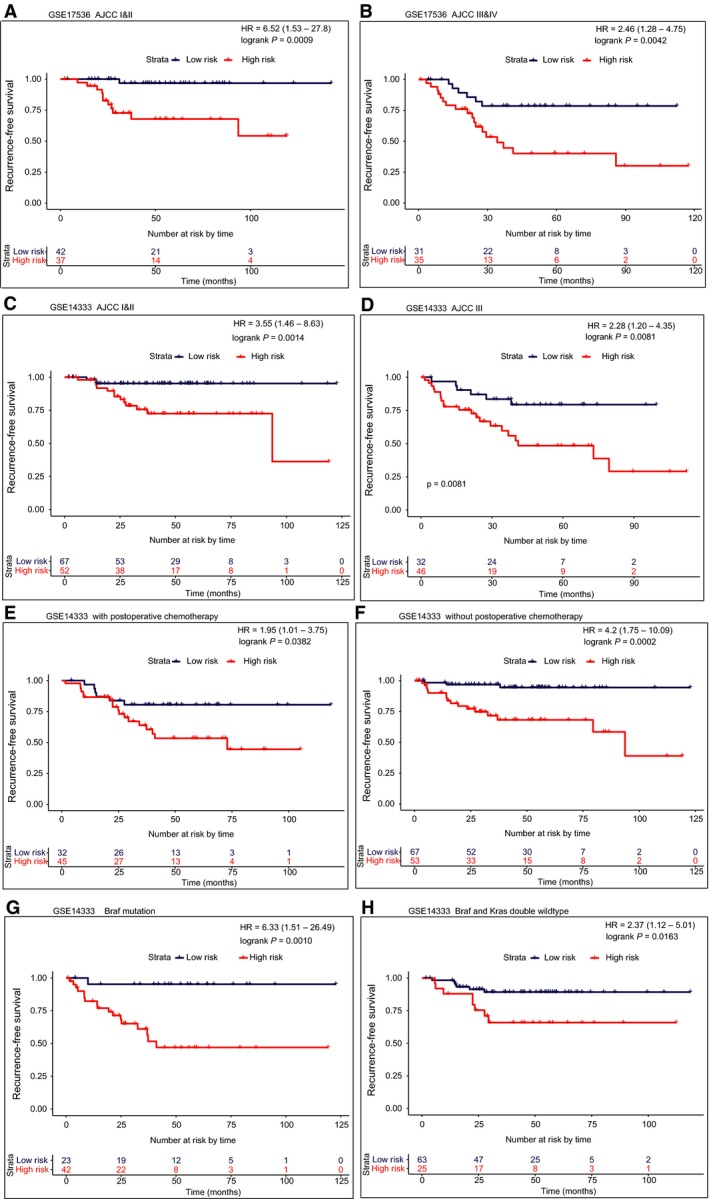
Kaplan–Meier survival analysis to evaluate the independence of the 13‐mRNA signature from AJCC stage, postoperative chemotherapy, and somatic muation. The patients from GSE62254 and GSE14333 were stratified into subgroups. The 13‐mRNA signature was applied to the low‐stage patients (A,C), high‐stage patients (B,D), patients with postoperative chemotherapy (E), patients without postoperative chemotherapy (F), patients with Braf gene mutation (G) or patients whose Kras gene and Braf genes were both wild type (H), separately.

To investigate whether the prognostic value of the 13‐mRNA signature was independent of tumor stage, the univariable and multivariable Cox regression analyses were performed using the risk score, age, gender, and tumor stage as covariates. We found that both the risk score and tumor stage were significantly associated with RFS even when adjusted by other clinical factors in GSE17536; there were no stage IV patients in GSE14333 (Fig. 2A–D). Then the stratification analysis was introduced based on tumor stage. Patients were stratified into two subgroups where AJCC stage I and II were fictitiously defined as early‐stage stratum and AJCC stage III and IV as late‐stage stratum. Regardless of the stratum, the 13‐mRNA signature still had the capability to distinguish high‐risk patients. Figure 3A showed that the prognosis of high‐risk patients was significantly worse than that of low‐risk patients in the early‐stage stratum of the GSE17536 cohort (HR = 6.52, 95% CI 1.53–27.8, *P *=* *0.0009), consistent with the results in the late‐stage stratum of the GSE17536 cohort (HR = 2.46, 95% CI 1.28–4.75, *P *=* *0.0042) (Fig. 3B). Stratification analysis of another dataset, GSE14333, yielded similar results; Fig. 3C shows the results in the early‐stage stratum of this cohort (HR = 3.55, 95% CI 1.46–8.63, *P *=* *0.0014), and Fig. 3D the results in the late‐stage (HR = 2.28, 95% CI 1.20–4.35, *P *=* *0.0081). These results indicate that the prognostic value of the 13‐mRNA signature was independent of tumor stage. We also assessed the prognostic ability of 13‐mRNA signature in patients based on postoperative chemotherapy, somatic mutation, and tumor location in dataset GSE14333. The patients were stratified into different subgroups, including patients with postoperative chemotherapy, patients without postoperative chemotherapy, patients with BRAF gene mutation, patients whose KRAS gene and BRAF gene were both wild‐type, patients with left‐sided CRC, patients with right‐sided CRC, and patients with rectum carcinoma. Interestingly, we found that except for the subgroup of rectum carcinoma, high‐risk patients in all the subgroups were inclined to have unfavorable RFS (Figs 3E–H, S4).

**Figure 4:**
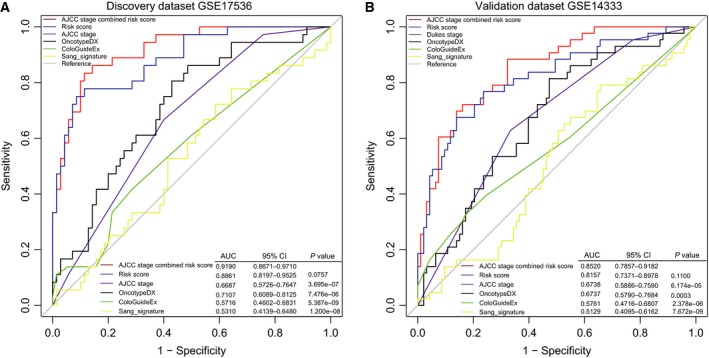
Receiver operating characteristic (ROC) analysis of the sensitivity and specificity of the recurrence prediction by the 13‐lncRNA risk score, tumor stage, oncotypeDX colon, ColoGuideEx, and Sang_signature in GSE17536 (*n *= 145) and GSE14333 (*n *= 197). *P*‐values were from the comparisons of the area under the ROC (AUC) of 13‐mRNA risk score combined with AJCC stage versus AUC of 13‐mRNA risk score, AJCC stage, oncotypeDX colon, ColoGuideEx, and Sang_signature separately.

We also performed ROC analysis to demonstrate the sensitivity and specificity of survival prediction in GSE17536 and GSE14333 sets. AUC was evaluated and compared between the 13‐mRNA risk score model and tumor stage. Figure 4A showed that the 13‐mRNA risk score model possessed a stronger predictive power than AJCC stage for the prognostic evaluation of CRC patients in the discovery cohort GSE17536 (0.8861 versus 0.6687, 95% CI 0.8197–0.9525 versus 0.5726–0.7647, *P *< 0.0001). When the 13‐mRNA risk score model was combined with tumor stage, no significant difference was found between the combined model and the 13‐mRNA risk score model (0.9190 versus 0.8861, 95% CI 0.8671–9710 versus 0.8197–0.9525, *P* = 0.0757). Analysis in the validation cohort GSE14333 produced similar results (Fig. 4B). The results from the validation dataset further confirmed the reliable predictive ability of the 13‐mRNA risk score model.

### 3.3 Construction of nomogram based on 13‐mRNA signature

**Figure 5:**
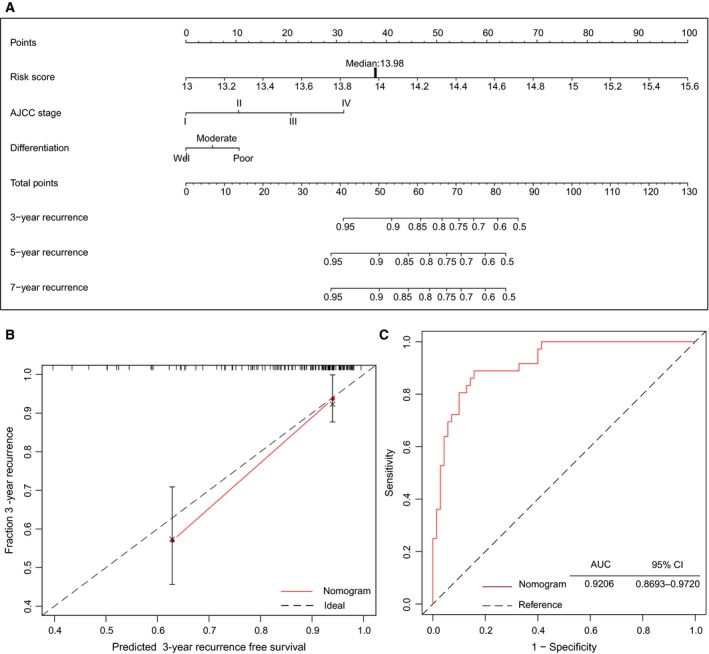
The nomogram to predict risk of cancer recurrence in GSE17536. (A) The nomogram for predicting proportion of patients with recurrence‐free survival. (B) The calibration plots for predicting recurrence at 3 years. Nomogram‐predicted probability of recurrence is plotted on the *x*‐axis; actual recurrence is plotted on the *y*‐axis. The solid line represents our nomogram and the vertical bars represent 95% CIs. (C) ROC curve based on the nomogram for recurrence probability.

To develop a practical method for clinicians to predict the probability of CRC recurrence, a nomogram was constructed which integrated the 13‐mRNA signature, tumor stage, and tumor differentiation (Fig. 5A). Figure 5B showed that the line‐segment in the calibration plots was very close to the 45° line which represented the best prediction, indicating that the nomogram did quite well. The predictive accuracy of the nomogram was calculated through ROC analysis: the AUC of nomogram is 0.9206, as shown in Fig. 5C.

### 3.4 Comparison with other known gene signatures

To further investigate the predictive power of the 13‐mRNA risk score model, some important gene signatures for prognosis prediction in CRC were analyzed in GSE17536 and GSE14333, including oncotypeDX colon cancer assay, ColoGuideEx, and Sang_signature (Agesen *et al*., 2012; Oh *et al*., 2012; Srivastava *et al*., 2014). Our study was not a comprehensive review of all biomarkers associated with CRC; the three selected signatures represented a purposive convenience sample. According to the associated formula, the prognostic indexes were calculated respectively (Table S5). We performed the univariable Cox regression analysis to investigate the association between each prognostic index and RFS, using the prognostic indexes as continuous variables. Figure S5 showed that except for the 13‐mRNA risk score model, only the oncotypeDX colon cancer assay was significantly associated with RFS. Moreover, the hazard ratio of the 13‐mRNA risk score model was significantly larger than that of oncotypeDX colon cancer assay. In the GSE17536 and GSE14333 datasets, the median RFS was 37.31 and 38.07 months, respectively. The patients whose follow‐up duration was less than median RFS were excluded if they still did not recur in the most recent follow‐up. ROC analysis was then performed (Fig. 4A,B); the AUC of the 13‐mRNA risk score model was significantly greater than that of other gene signatures. Remarkably, the selected gene signatures above mainly applied to prognosis predictions for stage II and III patients (Agesen *et al*., 2012; You *et al*., 2015). For a fair comparison, ROC analysis was carried out for stage II and III patients in dataset GSE17536, and yielded the similar results (Fig. S6), indicating that the 13‐mRNA risk score model outperformed other classifiers.

### 3.5 Identification of 13‐mRNA signature‐associated biological pathways

**Figure 6:**
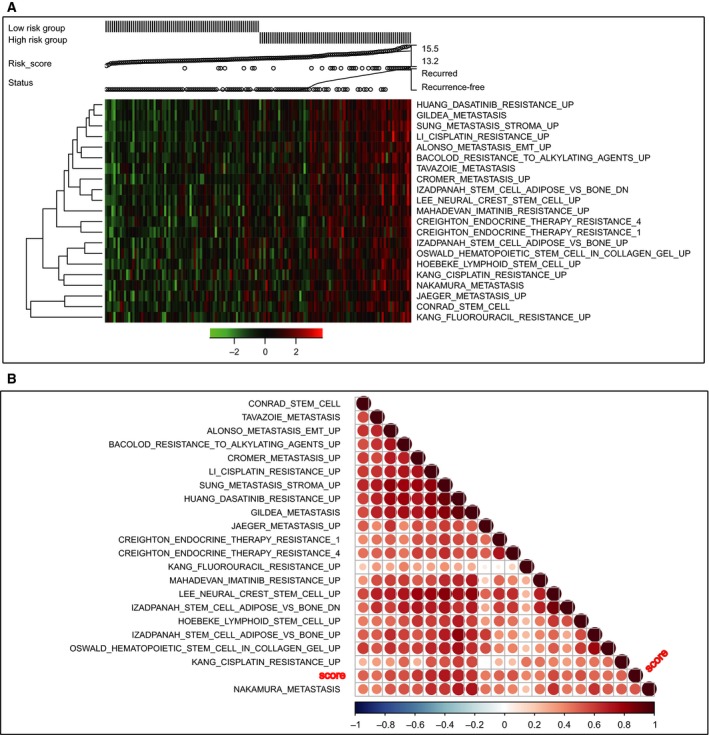
Pathway profiles across dataset GSE17536. Rows represent pathways, and columns represent patients. Each grid represents a score of pathway activity calculated by single‐sample GSEA. No further adjustment of the ssGSEA score was performed. The upper horizontal bar marked the information related to every patient, including the risk group, risk score (from low to high), and the recurrence status.

We performed ssGSEA analysis in dataset GSE17536 to identify the 13‐mRNA‐associated signaling pathways. The patients were divided into low‐ or high‐risk groups based on the 13‐mRNA model. Figure 6A showed that a group of pathways related to drug resistance, cancer metastasis, and stemness were significantly more activated in the high‐risk patients than low‐risk ones. Interestingly, these pathways and the risk score showed the same trend; with the increase of the risk score, the degree of enrichment gradually increased in the associated pathways. The association between the risk score and the pathways was further validated through correlation analysis, and the results confirmed the close relevance between them (*P* < 0.0001) (Fig. 6B, Table S6).

## 4 Discussion

Microarray profiling has received much attention in recent years, and has shown great promise in gaining an insight into molecular mechanisms through the analysis of thousands of genes (Cristescu *et al*., 2015; De Sousa *et al*., 2013). In our study, a 13‐mRNA signature was constructed for prognostic predication in CRC. When stratified by important clinicopathological factors, the 13‐mRNA signature retained a strong prognostic ability. Moreover, it outperformed other known gene signatures, indicating that it could improve the prediction of disease recurrence in CRC with considerable reliability and robustness.

For survival analysis, the Cox proportional hazards regression analysis is wide used at present. However, it is not suitable for high‐dimensional microarray data (Simon and Altman, 1994). Overfitting is one common limitation in modeling high‐dimensional microarray data for the selection of prognostic genes. The LASSO method could remove this limitation and was applied in our study for optimal selection of genes (Goeman, 2010; Tibshirani, 1997). Using this method, a 13‐mRNA signature was created by exploring the correlation between gene expression profiles and clinical outcome of CRC patients in the discovery dataset and was verified in two independent datasets, indicating favorable reproducibility.

The current TNM staging system (AJCC 6th edn) was closely associated with patient prognosis (O'Connell *et al*., 2004). The univariable and multivariable Cox regression analyses in our study consistently showed that tumor stage was a significant prognostic factor in the discovery and validation cohorts. Stratification analysis was therefore performed to investigate whether this 13‐mRNA signature was independent of tumor stage. The results showed that it could also discriminate the high‐risk patients from the stratified groups in the discovery and validation cohorts.

One important question should be mentioned here. The ethnicity in these three cohorts differed, as well as the constitution of tumor stages. Our discovery dataset (GSE17536) contained patients in all four AJCC stages; however, one validation dataset (GSE14333) had no AJCC stage IV patients; and another validation dataset (GSE33113) consisted only of AJCC stage II patients. The inconsistency in the constitution of tumor stages might increase the difficulty of validating our signature. The successful validation indicated that our gene signature was not only across populations, but was also independent of tumor stage, which was in accordance with the results of the stratification analysis above. As our signature was independent of tumor stage, it could be considered that the discrepancy in constitution of tumor stages did not affect the representativeness of these cohorts. Thus, the conclusions in our analyses were convincing.

Sporadic CRCs occurred through the accumulation of somatic genetic and epigenetic events (Carethers and Jung, 2015). Coincidentally, GSE14333 provided the information about KRAS and BRAF mutations, which were associated with a poor outcome in CRC. Moreover, the emergence of KRAS/NRAS mutations might underlie acquired resistance to target therapy in CRC (Dienstmann *et al*., 2015; Van Cutsem *et al*., 2011). Another important prognostic factor was adjuvant chemotherapy, which could significantly improve the outcome of CRC patients, especially for stage III patients (Ratkin, 1997); however, a study indicated that adjuvant FOLFOX for primary CRC was associated with a high frequency of somatic mutations in liver metastases and poor prognosis (Andreou *et al*., 2012). The interaction between these factors made it complex even for the prognostic prediction in CRC, necessitating further analysis to confirm the independence of our 13‐mRNA signature. Thus the CRC patients were also stratified into subgroups based on postoperative chemotherapy and somatic mutation. In accordance with the results above, the 13‐mRNA signature retained the ability to predict recurrence in all subgroups, indicating that this 13‐mRNA signature was independent of tumor stage, postoperative chemotherapy, and somatic mutation, and might complement clinicopathological features. Recent studies have shown that right‐ and left‐sided CRCs had different epidemiologic and histological characteristics, as well as underlying biologic mechanisms (Benedix *et al*., 2010; Bufill, 1990; Lee *et al*., 2017). However, when we stratified the patients by tumor location in GSE14333, we found that our 13‐mRNA signature could not discriminate high‐risk patients from the subgroup of rectum carcinoma. This result indicated that our signature may only apply to left‐sided or right‐sided CRCs. Note that there were only 23 patients with rectum carcinoma, so bias might have occurred in the stratification analysis. It is necessary to enlarge the sample size to generate more reliable results.

ROC analysis showed that our 13‐mRNA signature was superior to tumor stage for prognostic evaluation. To further improve the ability of prognostic prediction, we combined the 13‐mRNA risk model with tumor stage. There was no significant difference between the combined model and our gene signature, indicating that our 13‐mRNA signature could yield results by itself.

As a result of poor reproducibility, most established signatures have not been used clinically for prognostic prediction in CRC. The reasons of poor reproducibility are manifold. In early studies, small sample series and lack of validation in independent samples limited the strength of the conclusions. Besides, some gene signatures use too many genes for the construction of a model, which inhibits the clinical utility. Importantly, most studies of gene signatures are retrospective; the good reproducibility is still hampered by the lack of validation in prospective multicenter studies. To confirm further the availability of our 13‐mRNA signature, we chose three important gene signatures for comparison analysis in the discovery and validation datasets. Among them, both OncotypeDX colon cancer assay and ColoGuideEx have now been used clinically for CRC survival analysis (Agesen *et al*., 2012; O'Connell *et al*., 2010). As a new diagnostic test for determining the likelihood of recurrence in stage II colon cancer patients after surgical resection, OncotypeDX colon cancer assay has been commercially available worldwide since 2010 (Clark‐Langone *et al*., 2010; Webber *et al*., 2010). Specially, the effectiveness of the Oncotype DX colon cancer assay has been validated in a prospective multicenter study for the prediction of recurrence risk in stage II colon cancer patients (Brenner *et al*., 2016; Srivastava *et al*., 2014). Yothers *et al*. (2013) also found that incorporating the OncotypeDX colon cancer assay might better inform adjuvant therapy decisions in stage II and III colon cancer. Sang_signature could discriminate patient prognosis, as well as predict the response to adjuvant chemotherapy (Oh *et al*., 2012). Both Oncotype DX colon cancer assay and ColoGuideEx measured RFS risk as outcome, and Sang_signature used DFS, which was the same as RFS. So the three signatures were suitable for comparison with our signature. The results revealed that the 13‐mRNA signature was more significantly associated with RFS, and had more powerful ability for prognostic predication compared with the other gene signatures. Considering that Oncotype DX colon cancer assay and ColoGuideEx were more suitable for the stage II and III patients, these patients were selected for further comparison in discovery dataset GSE17536. Interestingly, our gene signature still significantly outperformed other gene signatures. The results indicated that this 13‐mRNA signature might be a useful tool for the management of CRC patients. As our study was retrospective, its reliability still needs further validation in a large prospective study.

As the 13‐mRNA signature showed considerable ability to discriminate the high‐risk patients based on risk score, the underlying molecular mechanism needs to be investigated. Studies revealed that cancer metastasis, drug resistance, and cancer stemness exerted an adverse impact on patient prognosis, and posed significant confusion among clinicians (Chau *et al*., 2004; Di Franco *et al*., 2014; Wicki *et al*., 2016). Coincidentally, the results of ssGSEA demonstrated that the 13‐mRNA signature was significantly associated with these pathways, which were highly enriched in the high‐risk group. A correlation analysis further confirmed this result, indicating these signaling pathways might underlie the carcinogenesis and progression of CRC, and providing a potential therapeutic target for clinic intervention.

The findings from ssGSEA analysis not only shed some light on the biogenesis of CRC, but also verified that it is reasonable to use our gene signature for prognostic prediction. The ssGSEA analysis demonstrated that our 13‐mRNA signature was closely associated with cancer metastasis, drug resistance, and cancer stemness, which are important prognostic factors (Chau *et al*., 2004; Di Franco *et al*., 2014; Wicki *et al*., 2016). Patients with these activated signaling pathways tend to have adverse outcomes. Thus using the 13‐mRNA signature for prognostic predication in CRC was quite logical and reliable. Meanwhile, the reason why our signature outperformed other signatures could perhaps be due partly to the appropriateness of our gene signature.

The biological functions of 13‐mRNA have been reported in previous research; however, only a few genes were investigated in CRC. THBS2 has been shown to function as a potent inhibitor of tumor growth and angiogenesis, and was associated with many kinds of diseases. Some studies indicated that it might be a biomarker for prognosis prediction of gastric and CRC (Jeong *et al*., 2015; Sun *et al*., 2014; Wang *et al*., 2016). CAV2 was a major component of the inner surface of caveolae, and the expression of CAV2 was necessary for the control of E2‐dependent cellular proliferation in breast cancer (Totta *et al*., 2016). SCG2 was a member of the chromogranin/secretogranin family of neuroendocrine secretory proteins, and it might contribute to the neuroendocrine differentiation by promoting the formation of secretory granules and the proliferation of prostate cancer cells (Courel *et al*., 2014). The SLC6A1 gene encoded a gamma‐aminobutyric acid (GABA) transporter, which removes GABA from the synaptic cleft (Hirunsatit *et al*., 2009), but there have been no studies in relation to cancer as of now. SAV is a scaffold protein containing a WW domain; SAV1 was reported to interact with HAX1 and attenuated its protective role against apoptosis in MCF‐7 breast cancer cells (Luo *et al*., 2011). MRPL35 encoded Mammalian mitochondrial ribosomal protein, whose impact on cancers has rarely been reported (Koc *et al*., 2001). SEZ6L2 encoded a seizure‐related protein, which was up‐regulated in lung cancer and was considered to be a novel prognostic marker (Ishikawa *et al*., 2006). ERO1A has previously been reported to be induced by hypoxia in cervical cancer cell lines (Halle *et al*., 2012), and it was considered to be a predictive biomarker in pancreatic ductal adenocarcinoma (Li *et al*., 2017). RAB3B was a member of the RAS oncogene family, which has been demonstrated to be closely implicated in CRC (Cha *et al*., 2016; Hoogwater *et al*., 2010). OBSL1 encoded a cytoskeletal adaptor protein, which was a member of the Unc‐89/obscurin family, and studies showed that 3M Syndrome was associated with this gene (Keskin *et al*., 2017; Marshall *et al*., 2015). CD109 encoded a glycosyl phosphatidylinositol‐linked glycoprotein, and some reports indicated it was concerned with the prognosis in CRC (Ashktorab *et al*., 2013; Yi *et al*., 2011). PTPN14 is a member of the protein tyrosine phosphatase family, and it has been reported to be a regulator of lymphatic function and choanal development (Au *et al*., 2010; Mendola *et al*., 2013). LRPAP1 interacts with the low density lipoprotein receptor‐related protein, and reports have shown that this gene was associated with myopia and Parkinson's disease (Khan *et al*., 2016; Singh *et al*., 2014). Up to now, most of the 13 mRNA have not been studied in CRC. Our study indicates that our method may be a new way to identify cancer‐associated genes. Studies on these prognostic genes might reveal new mechanisms underlying the carcinogenesis and development in CRC. In a word, the underlying molecular mechanism remains unclear, and the roles of these genes deserve further investigation in CRC.

Studies have shown that the benefit of adjuvant chemotherapy remains controversial in stage II CRC patients, which has created a great deal of difficulty for treatment in the clinic (O'Connell *et al*., 2004). Our signature possesses good power to discriminate high‐risk patients from low‐risk ones. Furthermore, pathway analysis indicated a close relation between our gene signature and drug resistance, and it therefore could help clinicians to assess the risk of recurrence and guide therapeutic regimens. In future studies, the ability of the 13‐mRNA model to assess the benefit of adjuvant chemotherapy deserves further investigation. To improve the utility in the clinic, we plan to validate our gene signature through RT‐PCR. RT‐PCR is much cheaper and easy to operate than gene microarray. This PCR‐based risk score method is the trend of the future, and will improve the management in CRC patients greatly.

The innovation of our research rests on the following aspects. First, the AUC of our 13‐mRNA signature is fairly large (> 0.8), indicating a good prognostic ability. Secondly, our study is a relatively systematic examination of prognostic gene signatures in CRC. Three representative gene signatures, including OncotypeDx, were selected for comparison analysis to verify further the prognostic power; our signature was demonstrated to be superior to all three. Thirdly, our study is of high methodological rationality. Our signature is derived from metastasis‐related mRNA, thus this signature is closely related to metastasis and should be suitable for prognostic assessment. The results of survival analyses are concordant with this hypothesis; in addition, pathway analysis confirms once again that our 13‐mRNA signature is closely associated with cancer metastasis.

There are some limitations to our study. First, one of our validation datasets, GSE33113, consisted only of stage II patients, which is not in agreement with the other two datasets. It is therefore not suitable for further analyses. Secondly, our study is retrospective and the sample size is limited, so the robustness and utility of the 13‐mRNA signature for prognostic prediction needs further validation in large prospective clinic trials, through which we can carry out a comprehensive evaluation of our signature. Thirdly, some of our analyses were hampered by a lack of detailed clinical information, which can be addressed in future through integrated data collection and detailed experimental design. Fourthly, there is still one more step to complete before clinical application. PCR‐based validation in large perspective trials will be of great clinical significance. Finally, more experimental data about these mRNA is required to elucidate the inherent association between the 13‐mRNA signature and CRC prognosis.

## 5 Conclusions

An innovative prognostic 13‐mRNA signature in CRC has been generated by exploring and analyzing the currently published microarray datasets. This 13‐mRNA signature is independent of tumor stage, postoperative chemotherapy, and somatic mutation. Moreover, it outperforms other known gene signatures, indicating that the 13‐mRNA signature may be a useful tool for clinicians and will facilitate personalized management of CRC patients.

## Data accessibility

Research data pertaining to this article is located at figshare.com: https://doi.org/10.6084/m9.figshare.5311105

## Author contributions

XT and XZ contributed equally to the work. XT drafted the manuscript. XZ and XT analyzed and interpreted all the data. TY, CY, and CS prepared the figures and tables. JH, HC, and JF reviewed and revised the manuscript. All authors approved the final manuscript.

## Supporting information


**Fig. S1.** Schematic diagram of work flow.

**Fig. S2.** Cross‐validation for tuning parameter selection in the LASSO model.

**Fig. S3.** 13‐mRNA risk score analysis of GSE17536.

**Fig. S4.** Kaplan–Meier survival analysis to evaluate the independence of the 13‐mRNA signature from tumor location.

**Fig. S5.** Forest plot summary of the analyses of prognostic classifiers in colorectal cancer (CRC).

**Fig. S6.** Receiver operating characteristic (ROC) analysis of the sensitivity and specificity of the recurrence prediction by the 13‐mRNA risk score, AJCC stage, prognostic indexes of oncotypeDX and ColoGuideEx in stage II and III patients of GSE17536 (*n *= 145).

**Table S1.** The raw clinic information data for the three datasets.

**Table S2.** Basic characteristics of patients in three datasets.

**Table S3.** Extra information on the three datasets.

**Table S4.** mRNA significantly associated with the recurrence‐free survival in the test series patients (*n *= 145).

**Table S5.** Calculation of prognostic indexes.

**Table S6.** Correlation analyses between risk score and associated pathways.
