# Supplementary material for: Recurrence‐associated gene signature optimizes recurrence‐free survival prediction of colorectal cancer
Source: Mol Oncol. 2017 Sep 23;11(11):1544–60. doi: 10.1002/1878-0261.12117 (PMC5664005; doi:10.1002/1878-0261.12117)
Supplement: Supplementary file 9 — Table S3. Extra information on the three datasets. [file MOL2-11-1544-s009.docx]

**Table S3: Extra information on three datasets**

**GSE17536 [**[**1**](#_ENREF_1)**]**

**Human Tissue and Microarray Platforms**

The protocols and procedures for this study were approved by the institutional review boards at the University of Alabama-Birmingham Medical Center, Vanderbilt Medical Center (VMC), the Veterans Administration Hospital (Nashville, TN), and the H. Lee Moffitt Cancer Center (MCC; Tampa, FL). Representative sections of fresh tissue specimens were flash frozen in liquid nitrogen and stored at −80°C until RNA isolation. Quality assessment slides were obtained to verify the diagnosis. Stage was assessed by American Joint Commission on Cancer guidelines. RNA was purified with the use of the RNeasy kit (QIAGEN, Valencia, CA). Mouse and human samples were hybridized to Affymetrix arrays (Mouse Genome 430 2.0 GeneChip Expression and Human Genome U133 Plus 2.0 GeneChip Expression Arrays, respectively; Santa Clara, CA).

**GSE14333 [**[**2**](#_ENREF_2)**]**

**Patients and gene expression microarray analysis**

Fresh-frozen tumor specimens from 293 consecutive CRC patients were retrieved from the tissue banks of the Royal Melbourne Hospital, Western Hospital and Peter MacCallum Cancer Center in Australia, and the H. Lee Moffitt Cancer Center in the United States; individuals who had received preoperative chemo-and/or radiotherapy or for whom tumor-derived total RNA was inadequate for microarray analysis (RIN < 6) were excluded. All patients gave informed consent, and this study was approved by the medical ethics committees of all sites. Patient median age at diagnosis was 67 years (range 26 to 92 years). All specimens were derived from primary carcinomas and were snap-frozen in liquid nitrogen immediately after surgery for storage at −80°C. Cases comprised 44 stage A, 95 stage B, 93 stage C and 61 stage D cancers; 252 were localized to the colon and 40 to the rectum, with one case missing this information. 22 of 94 patients who had stage B disease and 64 of 91 patients who had stage C disease had received standard adjuvant chemotherapy (either single agent 5-fluouracil/capecitabine or 5-fluouracil and oxaliplatin) or postoperative concurrent chemoradiotherapy (50.4 Gy in 28 fractions with concurrent 5- fluorouracil) according to hospital protocols. All patients were assessed annually. For stage B and C patients, follow-up and additional clinical data including patient gender and TNM staging were collected by Biogrid Australia 1 for Australian patients and the Moffitt Cancer Center Tumor Registry for US patients. The median duration of follow-up was 47.8 months (range 0.9 to 118.6 months) for the 140 patients without recurrence, and 19.1 months (range 1.6 to 93.7 months) for the 48 patients with local or distant recurrence. The median follow-up for all 188 patients was 37.2 months (range 0.9 to 118.6 months).

Total RNA was extracted using Trizol reagent (Invitrogen) from CRC samples containing >60% tumor cells. All samples included showed good integrity of 18S and 28S ribosomal bands (RIN > 6) using a 2100 Bioanalyzer (Agilent Technologies). Total RNA was labeled and hybridized to HG-U133Plus2.0 GeneChip arrays (Affymetrix) according to the manufacturer’s instructions. The microarray data on a subset of 174 tumors have been published previously (NCBI Gene Expression Omnibus, GSE5206 and GSE13067).

In addition, published gene expression data were retrieved for 42 stage A CRCs, 83 stage B, 73 stage C and 62 stage D CRCs analyzed as part of the Expression Project for Oncology (expO) 2 using HG-U133Plus2.0 GeneChip arrays (Affymetrix). Of the 62 stage D CRCs, 32 were primary cancer and 30 were metastectomy specimens.

None of the primary cancer patients had received preoperative therapy, but 17 metastectomy specimens were from patients who had received adjuvant chemotherapy treatment prior to resection. Data processing and analysis were performed using the statistical software package R and appropriate Bioconductor packages.

**GSE33113 [**[**3**](#_ENREF_3)**]**

**Clinical samples, microarray, K-Ras profile, and patient databases**

Tumor samples were collected of 90 patients with American Joint Committee on Cancer (AJCC) stage II CRC (AMC-AJCCII-90) as described previously in the work of de Sousa e Melo and colleagues. The study was approved for by the medical ethical board of the Academic Medical Center (AMC; Amsterdam, Netherlands) and in accordance with the rules and legislation on human experimentation in the Netherlands. Shortly, these patients with stage II CRC received surgery with curative intent in the AMC in Amsterdam, the Netherlands, between 1997 and 2006 (AMC-AJCCII-90). Extensive medical records and long-term follow-up are available for these patients. Fresh-frozen tissue was used to isolate total RNA from by TRIzol reagent (Invitrogen) according to the manufacturer's protocol. RNA was analyzed by NanoDrop ND-1000 and RNA 6000 Nano assay on the Agilent 2100 Bioanalyzer (Agilent Technologies). Affymetrix microarray analysis, fragmentation of RNA, labeling, hybridization to Human Genome U133 Plus 2.0 microarrays, and scanning were conducted in accordance with the manufacturer's protocol (Affymetrix). Microarray data can be viewed online (http://www.ncbi.nlm.nih.gov/geo/index.html) under Gene Expression Omnibus (GEO) accession number (GSE33113). Five patients were excluded for further analysis because CD133 expression levels were below detection threshold. The following probe sets, recognizing all isoforms of these proteins, were selected to avoid effects caused by differential splicing: 204304_s_at (CD133), 209771_x_at (CD24), 1553530_a_at (CD29), 210916_s_at (CD44), 201952_at (CD166), 213880_at (Lgr5), and 212224_at (ALDH1A1). The K-Ras profile was extracted from the study of Bild and colleagues. The following data sets were used: neuroblastoma, glioma (GSE4290), breast (GSE12276), and ovarian cancer (GSE12172).

References

1. Smith, J.J., et al., *Experimentally derived metastasis gene expression profile predicts recurrence and death in patients with colon cancer.* Gastroenterology, 2010. **138**(3): p. 958-68.

2. Jorissen, R.N., et al., *Metastasis-Associated Gene Expression Changes Predict Poor Outcomes in Patients with Dukes Stage B and C Colorectal Cancer.* Clin Cancer Res, 2009. **15**(24): p. 7642-7651.

3. Kemper, K., et al., *Mutations in the Ras-Raf Axis underlie the prognostic value of CD133 in colorectal cancer.* Clin Cancer Res, 2012. **18**(11): p. 3132-41.
