# Supplementary material for: Recurrence‐associated gene signature optimizes recurrence‐free survival prediction of colorectal cancer
Source: Mol Oncol. 2017 Sep 23;11(11):1544–60. doi: 10.1002/1878-0261.12117 (PMC5664005; doi:10.1002/1878-0261.12117)
Supplement: Supplementary file 10 — Table S4. mRNA significantly associated with the recurrence‐free survival in the test series patients (n = 145). [file MOL2-11-1544-s010.docx]

| Probe | Gene symbol | Coefficient | Description |
| --- | --- | --- | --- |
| 203083_at | THBS2 | 0.040757 | thrombospondin 2 |
| 203323_at | CAV2 | 0.077189 | caveolin 2 |
| 204035_at | SCG2 | 0.047062 | secretogranin II |
| 205152_at | SLC6A1 | 0.252962 | solute carrier family 6 (neurotransmitter transporter, GABA), member 1 |
| 218276_s_at | SAV1 | 0.306452 | salvador family WW domain containing protein 1 |
| 222775_s_at | MRPL35 | -0.06446 | mitochondrial ribosomal protein L35 |
| 223458_at | SEZ6L2 | 0.076798 | seizure related 6 homolog (mouse)-like 2 |
| 225750_at | ERO1A | 0.407432 | endoplasmic reticulum oxidoreductase 1 alpha |
| 227123_at | RAB3B | 0.22306 | member RAS oncogene family |
| 227574_at | OBSL1 | 0.49833 | obscurin-like 1 |
| 229900_at | CD109 | 0.038808 | CD109 molecule |
| 242321_at | PTPN14 | 0.015854 | protein tyrosine phosphatase, non-receptor type 14 |
| 242705_x_at | LRPAP1 | -0.04333 | low density lipoprotein receptor-related protein associated protein 1 |
|  |  |  |  |

Table S4. mRNAs significantly associated with the recurrence free survival in the test series patients (N=145)
