# Supplementary material for: Recurrence‐associated gene signature optimizes recurrence‐free survival prediction of colorectal cancer
Source: Mol Oncol. 2017 Sep 23;11(11):1544–60. doi: 10.1002/1878-0261.12117 (PMC5664005; doi:10.1002/1878-0261.12117)
Supplement: Supplementary file 11 — Table S5. Calculation of prognostic indexes. [file MOL2-11-1544-s011.docx]

### Table S5: Calculation of prognostic indexes

**Calculation of prognostic index for 13-mRNA signature**

The calculation of the risk score for individual patients was based on the multivariate model including all thirteen probes as shown in table S4. Firstly, through LASSO Cox regression analysis, we have identified 13 prognostic mRNAs and their associated coefficients. Then, multiply the expression level of each mRNA by its associated coefficient to generate the value for each mRNA. At last, sum all the values of 13 mRNAs to get the risk score for each patient. The formula was as follows:

Risk score = (0.040757*expression level of [THBS2](http://www.genecards.org/cgi-bin/carddisp.pl?gene=CHST9-AS1))+(0.077189* expression level of [CAV2](http://www.genecards.org/cgi-bin/carddisp.pl?gene=ENSG00000251538))+(0.047062* expression level of [SCG2](http://www.genecards.org/cgi-bin/carddisp.pl?gene=TPT1-AS1))+(0.252962* expression level of [SLC6A1](http://www.genecards.org/cgi-bin/carddisp.pl?gene=MIR100HG))+(0.306452* expression level of [SAV1](http://www.genecards.org/cgi-bin/carddisp.pl?gene=LOC400043))+(-0.06446* expression level of [MRPL35](http://www.genecards.org/cgi-bin/carddisp.pl?gene=LINC00340)) +(0.076798* expression level of [SEZ6L2](http://www.genecards.org/cgi-bin/carddisp.pl?gene=LOC283174)) +(0.407432* expression level of [ERO1A](http://www.genecards.org/cgi-bin/carddisp.pl?gene=LOC100133985)) +(0.22306* expression level of [RAB3B](http://www.ncbi.nlm.nih.gov/UniGene/clust.cgi?ORG=Hs&CID=93194)) +(0.49833* expression level of [OBSL1](http://www.genecards.org/cgi-bin/carddisp.pl?gene=LOC401093))+ (0.038808* expression level of [CD109](http://www.genecards.org/cgi-bin/carddisp.pl?gene=ENSG00000233236)) + (0.015854* expression level of [PTPN14](http://www.genecards.org/cgi-bin/carddisp.pl?gene=ENSG00000229565)) + (-0.04333* expression level of [LRPAP1](http://www.genecards.org/cgi-bin/carddisp.pl?gene=ENSG00000229565)).

**Algorithms to calculate risk scores for OncotyeDX, ColoGuideEx, and Sang_signature**

1. OncotyeDX colon cancer assay

Recurrence Risk score is calculated using the prespecified genes and algorithm [[1](#_ENREF_1), [2](#_ENREF_2)]:

The O'Connell Recurrence Risk (RS) score is composed of 12 genes among which 5 reference genes and 7 genes associated to recurrence.

For the reference genes, when several probe set were possible, the less variant one was selected.

For the other genes, data were median gene centered and aggregated by mean if several probe sets were available.

Then the recurrence genes intensities for each sample were subtracted by the mean of the reference gene per sample and the formula was applied for each sample. RSu = 0·15* mean(BGN,FAP,INHBA)-0·3*mean(MKI67,MYC,MYBL2)+ 0·15* GADD45B)

This score was then rescaled RS=44*(RSu+0.82).

2. ColoGuideEx

ColoGuideEx: a robust gene classifier specific for stage II colorectal cancer prognosis [[3](#_ENREF_3)].

**Table 1**: Identities of the 13 genes in the prognostic expression signature and their univariate association with prognosis

| Transcript cluster ID[^*^](http://gut.bmj.com/content/61/11/1560/T1.expansion.html#fn-2) | Gene symbol[^†^](http://gut.bmj.com/content/61/11/1560/T1.expansion.html#fn-3) | Gene name[^†^](http://gut.bmj.com/content/61/11/1560/T1.expansion.html#fn-3) | Chromosome location[^†^](http://gut.bmj.com/content/61/11/1560/T1.expansion.html#fn-3) | Gene expression level and association with poor prognosis |
| --- | --- | --- | --- | --- |
| 2453006 | PIGR | Polymeric immunoglobulin receptor | 1q31-q41 | Low |
| 2732508 | CXCL13 | Chemokine (C-X-C motif) ligand 13 | 4q21 | Low |
| 3388830 | MMP3 | Matrix metallopeptidase 3 (stromelysin 1, progelatinase) | 11q22.3 | Low |
| 3453732 | TUBA1B | Tubulin, alpha 1b | 12q13.12 | Low |
| 2968652 | SESN1 | Sestrin 1 | 6q21 | High |
| 3063589 | AZGP1 | alpha-2-Glycoprotein 1, zinc-binding | 7q22.1 | High |
| 3868768 | KLK6 | Kallikrein-related peptidase 6 | 19q13.3 | High |
| 2965206 | EPHA7 | EPH receptor A7 | 6q16.3 | High |
| 3059464 | SEMA3A | Sema domain, immunoglobulin domain (Ig), short basic domain, secreted, (semaphorin) 3A | 7p12.1 | High |
| 3802924 | DSC3 | Desmocollin 3 | 18q12.1 | High |
| 2773958 | CXCL10 | Chemokine (C-X-C motif) ligand 10 | 4q21 | Low |
| 2925871 | ENPP3 | Ectonucleotide pyrophosphatase/phosphodiesterase 3 | 6q22 | High |
| 3314040 | BNIP3 | BCL2/adenovirus E1B 19kDa interacting protein 3 | 10q26.3 | High |

- [↵](http://gut.bmj.com/content/61/11/1560/T1.expansion.html#xref-fn-2-1)***** Affymetrix GeneChip Human Exon 1.0 ST Arrays transcript cluster ID.
- [↵](http://gut.bmj.com/content/61/11/1560/T1.expansion.html#xref-fn-3-1)**†** Approved by the HUGO Gene Nomenclature Committee (8 March 2011).

 The ranking of the combinations of gene expression signatures revealed that at least five genes with a positive prognostic score were needed to separate the patients with a low risk of relapse from those with a high risk of relapse. Poor prognosis was associated with low expression of the gene PIGR, CXCL13, MMP3,TUBA1B and CXCL10 genes  and high expression of SESN1, AZGP1, KLK6, EPHA7, SEMA3A, DSC3,ENPP3 and BNIP3.

Stage II CRC samples within each series were categorized into a low or high risk of relapse group based on the number of genes exceeding the 80th and 20th percentile levels of each gene in the signature.

3. Sang_signature

The prediction rule is defined by the inner sum of the weights (wi) and expression (xi) of genes[[4](#_ENREF_4)].

A sample is classified to the class A if the sum is greater than the threshold; that is, Σiwi xi > threshold. The thresholds for the Compound covariate predictor are -65 for 80-probe model and -125 for 114-probe model.

| Affy Probe ID | Gene weight by CCP |
| --- | --- |
| 1554436_a_at | ‐4.9862 |
| 204855_at | ‐6.4427 |
| 223447_at | ‐4.4909 |
| 204014_at | ‐8.1565 |
| 204748_at | ‐6.8569 |
| 202437_s_at | ‐6.0069 |
| 217428_s_at | ‐6.4194 |
| 238017_at | ‐5.4433 |
| 1555745_a_at | ‐4.6068 |
| 205625_s_at | ‐4.0489 |
| 205927_s_at | ‐6.0357 |
| 218804_at | ‐8.0138 |
| 205844_at | ‐5.9672 |
| 209792_s_at | ‐5.9919 |
| 205626_s_at | ‐3.7526 |
| 209875_s_at | ‐6.9299 |
| 223122_s_at | ‐5.929 |
| 204457_s_at | ‐7.7531 |
| 214974_x_at | ‐3.921 |
| 226545_at | ‐8.1108 |
| 213994_s_at | ‐7.5094 |
| 215446_s_at | ‐8.0178 |
| 221872_at | ‐6.6738 |
| 206392_s_at | ‐6.4033 |
| 210145_at | ‐5.2604 |
| 223121_s_at | ‐6.5188 |
| 210004_at | ‐7.9029 |
| 206391_at | ‐6.8073 |
| 203820_s_at | ‐4.1619 |
| 204446_s_at | ‐8.3325 |
| 219410_at | ‐9.1399 |
| 223642_at | ‐3.8279 |
| 37892_at | ‐5.9116 |
| 1554997_a_at | ‐6.0288 |
| 201926_s_at | ‐7.4163 |
| 207480_s_at | ‐7.5817 |
| 211506_s_at | ‐5.4041 |
| 229271_x_at | ‐7.0502 |
| 209406_at | ‐5.7809 |
| 1555778_a_at | ‐7.5524 |
| 205081_at | ‐6.2691 |
| 1555950_a_at | ‐7.1867 |
| 204298_s_at | ‐8.1004 |
| 219508_at | ‐4.9778 |
| 201939_at | ‐7.2579 |
| 204006_s_at | ‐6.7545 |
| 228708_at | ‐5.3538 |
| 202952_s_at | ‐7.9813 |
| 217028_at | ‐7.735 |
| 228241_at | ‐3.5661 |
| 201925_s_at | ‐7.3453 |
| 205513_at | ‐3.783 |
| 206025_s_at | ‐7.6183 |
| 226777_at 1. | ‐7.0163 |
| 1554018_at | ‐6.8041 |
| 201012_at | ‐7.3506 |
| 204015_s_at | ‐6.7619 |
| 206026_s_at | ‐6.9589 |
| 209955_s_at | ‐8.2826 |
| 226517_at | ‐8.0456 |
| 205941_s_at | ‐6.0911 |
| 202388_at | ‐6.9578 |
| 32128_at | ‐4.6637 |
| 202286_s_at | ‐2.9026 |
| 212992_at | ‐6.2249 |
| 227399_at | ‐6.6893 |
| 202436_s_at | ‐5.4279 |
| 207814_at | 4.8125 |
| 242414_at | 10.707 |
| 205983_at | 7.0126 |
| 226654_at | 6.6162 |
| 228915_at | 6.5282 |
| 209460_at | 9.5403 |
| 231426_at | 8.0176 |
| 209425_at | 9.4987 |
| 222696_at | 9.3679 |
| 213385_at | 8.0754 |
| 203896_s_at | 7.8312 |
| 207412_x_at | 10.856 |
| 224221_s_at | 7.6381 |
| 225016_at | 7.4784 |
| 205043_at | 9.5616 |
| 208121_s_at | 7.0989 |
| 220376_at | 7.2744 |
| 222257_s_at | 7.8656 |
| 235147_at | 9.4303 |
| 219962_at | 6.7967 |
| 211207_s_at | 8.1442 |
| 218963_s_at | 6.5891 |
| 204044_at | 10.137 |
| 241547_at | 11.005 |
| 206418_at | 7.5343 |
| 218806_s_at | 10.438 |
| 219471_at | 10.398 |
| 203895_at | 8.2417 |
| 232136_s_at | 9.6193 |
| 218807_at | 10.999 |
| 232737_s_at | 8.1877 |
| 205799_s_at | 7.6656 |
| 206000_at | 9.3252 |
| 206286_s_at | 11.47 |
| 229215_at | 11.477 |
| 209459_s_at | 9.6781 |
| 229725_at | 8.3602 |
| 44790_s_at | 10.076 |
| 205767_at | 6.6868 |
| 205892_s_at | 6.4933 |
| 219948_x_at | 7.4143 |
| 206143_at | 5.9042 |
| 207457_s_at | 11.236 |
| 227735_s_at | 10.769 |
| 227736_at | 10.695 |
| 205910_s_at | 10.44 |
| 1553970_s_at | 10.44 |

**References**

1. Yothers, G., et al., *Validation of the 12-gene colon cancer recurrence score in NSABP C-07 as a predictor of recurrence in patients with stage II and III colon cancer treated with fluorouracil and leucovorin (FU/LV) and FU/LV plus oxaliplatin.* J Clin Oncol, 2013. **31**(36): p. 4512-9.

2. Gray, R.G., et al., *Validation study of a quantitative multigene reverse transcriptase-polymerase chain reaction assay for assessment of recurrence risk in patients with stage II colon cancer.* J Clin Oncol, 2011. **29**(35): p. 4611-9.

3. Agesen, T.H., et al., *ColoGuideEx: a robust gene classifier specific for stage II colorectal cancer prognosis.* Gut, 2012. **61**(11): p. 1560-7.

4. Oh, S.C., et al., *Prognostic gene expression signature associated with two molecularly distinct subtypes of colorectal cancer.* Gut, 2012. **61**(9): p. 1291-8.
