# Supplementary material for: Recurrence‐associated gene signature optimizes recurrence‐free survival prediction of colorectal cancer
Source: Mol Oncol. 2017 Sep 23;11(11):1544–60. doi: 10.1002/1878-0261.12117 (PMC5664005; doi:10.1002/1878-0261.12117)
Supplement: Supplementary file 12 — Table S6. Correlation analyses between risk score and associated pathways. [file MOL2-11-1544-s012.docx]

Table S6: Correlation analysis between risk score and associated pathways

| Pathways | correlation coefficient | p-value | 95%CI |
| --- | --- | --- | --- |
| SUNG_METASTASIS_STROMA_UP | 0.7109 | < 2.2E-16 | 0.6197-0.7832 |
| HUANG_DASATINIB_RESISTANCE_UP | 0.6654 | < 2.2E-16 | 0.5635-0.7474 |
| CROMER_METASTASIS_UP | 0.6134 | 2.38E-16 | 0.5004-0.7058 |
| GILDEA_METASTASIS | 0.6156 | < 2.2E-16 | 0.5031-0.7076 |
| LI_CISPLATIN_RESISTANCE_UP | 0.6494 | < 2.2E-16 | 0.5440-0.7346 |
| ALONSO_METASTASIS_EMT_UP | 0.5626 | 1.77E-13 | 0.4400-0.6647 |
| LEE_NEURAL_CREST_STEM_CELL_UP | 0.5554 | 4.18E-13 | 0.4314-0.6587 |
| NAKAMURA_METASTASIS | 0.5154 | 3.28E-11 | 0.3847-0.6258 |
| HOEBEKE_LYMPHOID_STEM_CELL_UP | 0.5352 | 4.05E-12 | 0.4078-0.6422 |
| CONRAD_STEM_CELL | 0.4866 | 5.44E-10 | 0.3515-0.6019 |
| BACOLOD_RESISTANCE_TO_ALKYLATING_AGENTS_UP | 0.5381 | 2.96E-12 | 0.4111-0.6446 |
| MAHADEVAN_IMATINIB_RESISTANCE_UP | 0.4253 | 9.71E-08 | 0.2818-0.5502 |
| IZADPANAH_STEM_CELL_ADIPOSE_VS_BONE_DN | 0.4739 | 1.73E-09 | 0.3370-0.5913 |
| IZADPANAH_STEM_CELL_ADIPOSE_VS_BONE_UP | 0.4762 | 1.42E-09 | 0.3395-0.5931 |
| CREIGHTON_ENDOCRINE_THERAPY_RESISTANCE_1 | 0.4931 | 2.96E-10 | 0.3589-0.6073 |
| CREIGHTON_ENDOCRINE_THERAPY_RESISTANCE_4 | 0.5301 | 7.07E-12 | 0.4018-0.6380 |
| TAVAZOIE_METASTASIS | 0.4630 | 4.51E-09 | 0.3245-0.5821 |
| KANG_FLUOROURACIL_RESISTANCE_UP | 0.3978 | 7.24E-07 | 0.2511-0.5267 |
| OSWALD_HEMATOPOIETIC_STEM_CELL_IN_COLLAGEN_GEL_UP | 0.4649 | 3.84E-09 | 0.3266-0.5837 |
| JAEGER_METASTASIS_UP | 0.4271 | 8.43E-08 | 0.2839-0.5517 |
| KANG_CISPLATIN_RESISTANCE_UP | 0.4857 | 5.93E-10 | 0.3504-0.6011 |
|  |  |  |  |
