# Supplementary material for: Recurrence‐associated gene signature optimizes recurrence‐free survival prediction of colorectal cancer
Source: Mol Oncol. 2017 Sep 23;11(11):1544–60. doi: 10.1002/1878-0261.12117 (PMC5664005; doi:10.1002/1878-0261.12117)
Supplement: Supplementary file 13 [file MOL2-11-1544-s013.docx]

**Table S1.** The raw clinic information data for the three datasets.

**Table S2.** Basic characteristics of patients in three datasets.

**Table S3.** Extra information on the three datasets.

**Figure S1.** Schematic diagram of work flow. The orders of analyses to generate the risk mode and assess prognostic value of mRNA signature in CRC.

**Figure S2.** Cross-validation for tuning parameter selection in the LASSO model. The red dotted line represents the cross-validation curve. The solid vertical lines are partial likelihood deviance standard error (SE). The dotted vertical lines are drawn at the optimal values by minimum criteria and 1 SE criteria. We plotted the partial likelihood deviance versus log (lambda), where the tuning parameter is.

**Table S4.** mRNA significantly associated with the recurrence-free survival in the test series patients (*n*= 145)

**Table S5.** Calculation of prognostic indexes

**Figure S3.** 13-mRNA risk score analysis of GSE17536. The distribution of 13-mRNA Z-score transformed risk score, patients’ recurrence status and mRNA expression signature were analyzed in the GSE17536 series patients (*n*= 145). (A) Risk score distribution. (B) Patients’ recurrence status and time. (C) Heatmap of the mRNA expression profiles.

**Figure S4.** Kaplan–Meier survival analysis to evaluate the independence of the 13-mRNA signature from tumor location. The patients from GSE14333 were stratified into three subgroups. The 13-mRNA signature was applied to the patients with left-sided CRC (A), patients with right-sided CRC (B), and patients with rectum carcinoma (C), separately.

**Figure S5.** Forest plot summary of the analyses of prognostic classifiers in colorectal cancer (CRC). Univariable analyses of the 13-mRNA risk score, oncotypeDX colon, ColoGuideEx, and Sang_signature to investigate the association between each prognostic index and RFS using the prognostic indexes as continuous variables on GSE17536 (A) and GSE14333 (B) datasets. The blue squares on the transverse lines represent the hazard ratio (HR), and the red transverse lines represent 95% CI.

**Figure S6.** Receiver operating characteristic (ROC) analysis of the sensitivity and specificity of the recurrence prediction by the 13-mRNA risk score, AJCC stage, prognostic indexes of oncotypeDX and ColoGuideEx in stage II & III patients of GSE17536 (*n*= 145). *P*-values were from the comparisons of the area under the ROC (AUC) of 13-mRNA risk score combined with AJCC stage versus AUC of 13-mRNA risk score, AJCC stage, prognostic indexes of oncotypeDX and ColoGuideEx, separately.

**Table S6.** Correlation analyses between risk score and associated pathways.
